# Gut microbiota assemblages of generalist predators are driven by local- and landscape-scale factors

**DOI:** 10.3389/fmicb.2023.1172184

**Published:** 2023-05-15

**Authors:** Hafiz Sohaib Ahmed Saqib, Linyang Sun, Gabor Pozsgai, Pingping Liang, Mohsan Ullah Goraya, Komivi Senyo Akutse, Minsheng You, Geoff M. Gurr, Shijun You

**Affiliations:** ^1^State Key Laboratory for Ecological Pest Control of Fujian and Taiwan Crops, Institute of Applied Ecology, Fujian Agriculture and Forestry University, Fuzhou, China; ^2^Guangdong Provincial Key Laboratory of Marine Biology, College of Science, Shantou University, Shantou, China; ^3^Joint International Research Laboratory of Ecological Pest Control, Ministry of Education, Fuzhou, China; ^4^Ministerial and Provincial Joint Innovation Centre for Safety Production of Cross-Strait Crops, Fujian Agriculture and Forestry University, Fuzhou, China; ^5^Ce3C - Centre for Ecology, Evolution and Environmental Changes, Azorean Biodiversity Group, CHANGE – Global Change and Sustainability Institute, University of the Azores, Faculty of Agricultural Sciences and Environment, Angra do Heroísmo, Açores, Portugal; ^6^Center for Infection and Immunity, Guangdong Provincial Engineering Research Center of Molecular Imaging, Guangdong Provincial Key Laboratory of Biomedical Imaging, The Fifth Affiliated Hospital, Sun Yat-Sen University, Zhuhai, Guangdong, China; ^7^Guangdong Provincial Key Laboratory of Infectious Diseases and Molecular Immunopathology, Shantou University Medical College, Shantou, China; ^8^Plant Health Theme, International Centre of Insect Physiology and Ecology, Nairobi, Kenya; ^9^Gulbali Institute, Charles Sturt University, Orange, NSW, Australia

**Keywords:** agroecosystem, microbiome, high-throughput sequencing, microbe-environment interactions, Lycosidae

## Abstract

The gut microbiomes of arthropods have significant impact on key physiological functions such as nutrition, reproduction, behavior, and health. Spiders are diverse and numerically dominant predators in crop fields where they are potentially important regulators of pests. Harnessing spiders to control agricultural pests is likely to be supported by an understanding of their gut microbiomes, and the environmental drivers shaping microbiome assemblages. This study aimed to deciphering the gut microbiome assembly of these invertebrate predators and elucidating potential implications of key environmental constraints in this process. Here, we used high-throughput sequencing to examine for the first time how the assemblages of bacteria in the gut of spiders are shaped by environmental variables. Local drivers of microbiome composition were globally-relevant input use system (organic production vs. conventional practice), and crop identity (Chinese cabbage vs. cauliflower). Landscape-scale factors, proportion of forest and grassland, compositional diversity, and habitat edge density, also strongly affected gut microbiota. Specific bacterial taxa were enriched in gut of spiders sampled from different settings and seasons. These findings provide a comprehensive insight into composition and plasticity of spider gut microbiota. Understanding the temporal responses of specific microbiota could lead to innovative strategies development for boosting biological control services of predators.

## 1. Introduction

Modern DNA-based methods have revealed that the guts of arthropods harbor a wide variety of microbes exerting strong effects on host fitness, including development, reproduction, host nutrition, stress tolerance against biotic and abiotic stresses, and regulation of host-pathogen interactions (Engel and Moran, 2013; Jang and Kikuchi, 2020). There is a strong research focus on bacterial diversity within arthropods recently focused on microbial communities' interactions with their hosts. For example, Wu et al. (2021) demonstrated that the gut microbiota of spiders have the ability to modulate the utilization of polysaccharides as a source of energy. These findings indicate that associated microbes may have significant implications in various aspects of spider biology, including digestion, immunity, silk production, and behavior.

Spiders are one of the most abundant and diverse groups of predators in agricultural fields, and exhibit a diversity of foraging, hunting, morphological and physiological traits that allow several closely related species to coexist (Viera and Gonzaga, 2017). These substantial evolutionary adaptations contribute to niche differentiation (Michalko et al., 2016) including the utilization of diverse prey. These factors allow spiders to persist in and exploit a wide range of spatio-temporal habitats including in-crop residency whilst utilizing non-pest prey prior to “switching” when crop pests become available (Rand et al., 2006). Since gut microbial communities of arthropods are affected by the hosts' diet (Marinozzi et al., 2013), the effects of diet-related environmental factors on the identity and composition of spider gut microbiota are likely to be significant, albeit largely unknown.

The gut microbiota of arthropods also varies geographically (Krawczyk et al., 2022), suggesting a role of factors in the surrounding environment such as microbes in locally available diet or foraging substrates. Spiders utilize a wide variety of prey located in contrasting habitat types such as foliage or the soil surface and, since the surrounding landscape composition drives the abundance and diversity of these food items (Saqib et al., 2021), it is plausible that spiders' microbial communities can be affected by such factors. Effects on gut microbial diversity of arthropods from the surrounding habitat have been reported (Tiede et al., 2017). However, the effects of larger, landscape-scale effects, along with local factors such as farming practices, remain largely unclear.

Predator and prey species coexist in an environment full of toxins (such as plant defense compounds, pollutants, and pesticides). Pesticides are widely used in conventional farming systems, which results in evolutionary adaptations in both predators and prey to counteract their effects (e.g., the mechanism of detoxification, changes in the target site, neutralization of toxins) (Köhler and Triebskorn, 2013). Although it is believed that all these resistance mechanisms are encoded within the insect genomes, omics analyses have revealed that a variety of organisms possess gut microbes, that actively degrade toxins including plant defense and pesticidal compounds (Itoh et al., 2018). Therefore, farming systems with varying pesticide use may actively shape invertebrates' gut microbiota which, in turn, may increase pesticide resistance and thus fitness.

In brassica vegetable growing systems, different species of *Brassica* are often cultivated in close proximity and such polycultures can affect the diversity of prey species available to predators (Brandmeier et al., 2021; N'Woueni and Gaoue, 2022). Furthermore, the prey assemblages vary with the changing cropping patterns and climate of different seasons (Liu et al., 2018; Radzikowski et al., 2020). Since the composition of the predator gut microbiome may be affected by diet, including potential acquisition of microbes from prey, a range of environmental drivers is likely influencing the spider microbiomes. Studies have shown that associated microbes within the host can be acquired either from the external environment or through vertical transmission from other organisms (Hauke and Paul, 2011; Kwong et al., 2022). Understanding the environmental factors that determine the composition of gut microbes may thus provide insights into a, so far unexplored, segment of ecological interactions.

Few studies have been reported of the gut microbes of spiders (Vanthournout and Hendrickx, 2015; Zhang et al., 2017, 2018; Ng et al., 2018; Kumar et al., 2020; Tyagi et al., 2021), despite the foregoing range of factors that are likely to be drivers of spider performance as control agents of pests in crop systems. Accordingly, we employed 16S rDNA high-throughput sequencing to characterize the gut microbial assemblages of spiders sampled from brassica vegetable fields and evaluate the effects of local- and landscape-scale variables on taxonomic composition. Especially, we hypothesized that the gut microbiome is highly plastic rather than fixed, with seasons, local agronomic factors and the wider landscape significantly influencing the assemblages of microbes in the gut of spiders. Furthermore, we hypothesized that specific farming practices may influence the assemblages of bacterial communities that promote resistance to pesticides.

## 2. Material and methods

### 2.1. Study system, area, and design

**Figure 1:**
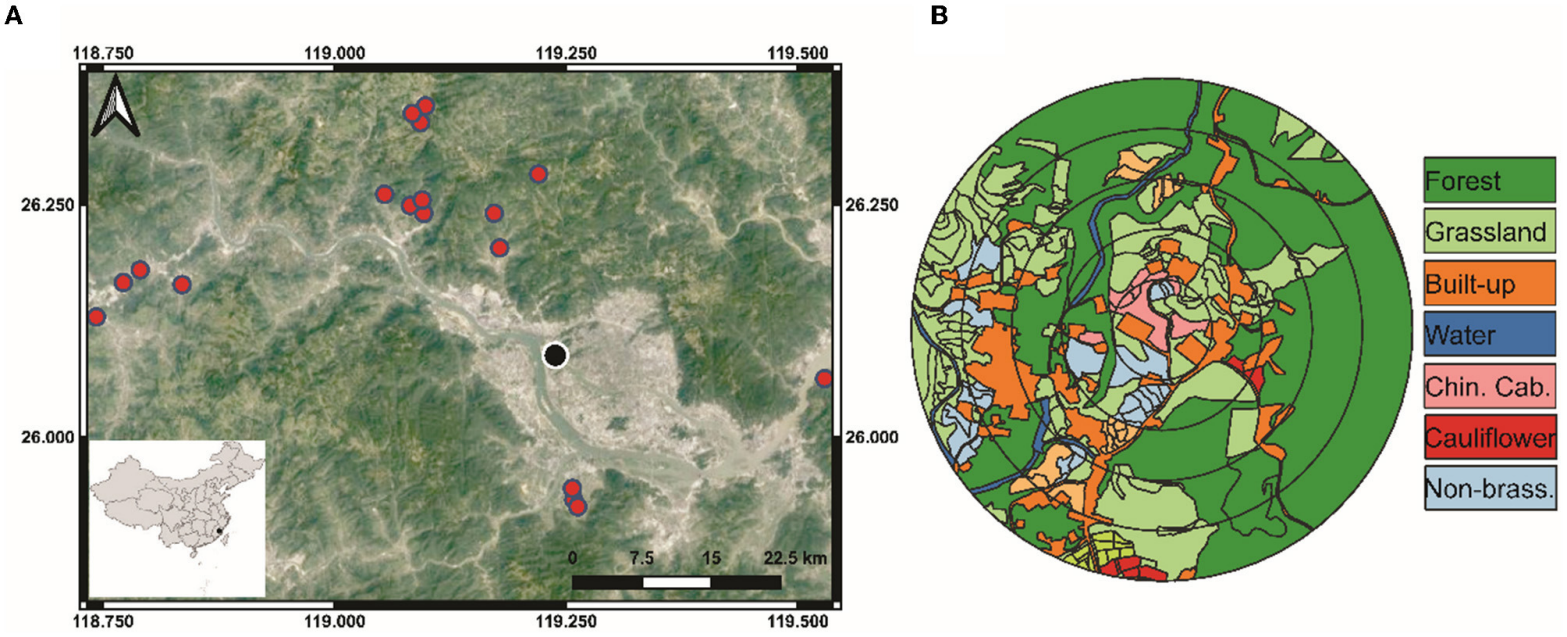
Map showing the **(A)** sampling locations in Fujian Province, southeastern China. **(B)** Example of landscape mapping of different habitats within 100, 200, 300, 400, and 500 m radius buffers around the focal sampling field.

In 2019, we sampled spiders from vegetable farms in Fujian Province, southeastern China, once every season for the four seasons (i.e., four times) at crop maturity (Figure 1A). In this region, farms are typically smallholdings with highly dynamic, polyculture vegetable production systems. A total of 18 commercial crop fields were chosen, each at least 1 km apart, to represent various management systems and crop types, as well as varying fractions of land usage in the surrounding landscapes (Figure 1B). On all fields, either Chinese cabbage (*Brassica rapa pekinensis*) or cauliflower (*Brassica oleracea*) were grown, using direct sowing and seedling transplanting methods.

Fields were divided into two groups based on management practices: 12 conventionally managed (i.e., synthetic pesticides or fertilizers were used) and six organically managed (i.e., no synthetic pesticides or fertilizers were used). Since samples were collected from farmers' fields, we did not interfere or control the inputs in either conventional or organic farms nor did we intervene in any management practices. The disparity in field count between these two farming groups reflected their relative representation in this region. In all the four seasons, statistically adequate replicates of Chinese cabbage and cauliflower were represented in both organic and conventionally managed fields.

To ensure that the crops were not damaged and prevent surface DNA contamination, spiders were hand-collected using sampling method 4 described of Sørensen et al. (2002) by two people for an hour of active searching per site. Only spiders belonging to the Lycosidae family were collected since these were numerically dominant and known to be potentially important predators of brassica pests (Mabin et al., 2020; Cuff et al., 2022). Individual lycosids were sampled from the soil surface and plants directly into 5 mL clean tubes. Samples were immediately transported to the laboratory in an icebox, and then kept at −80°C.

### 2.2. Landscape analyses

A drone (PHANTOM 4, Shenzhen Dajiang Baiwang Technology Co., Ltd., China) was used to take aerial photographs of each field and the landscape to a 500 m radius circle to investigate the distribution of habitats. Aerial images were used to classify the vegetation types within the 500 m circles into grassland, forest, built-up (e.g., residential land, greenhouses, and roads), water surfaces (e.g., streams and ponds), Chinese cabbage, cauliflower, other Brassica crops (e.g., broccoli, canola, and mustard), non-brassica crops (e.g., pepper, eggplant, corn, and beans) and fallow land (arable having no crop) (Figure 1B). The compositional diversity of landscape was assessed by calculating the Shannon-Wiener diversity index (SHDI). QGIS 3.4 was used to calculate the proportions of various habitat types and edge densities in the 500-meter radius landscape surrounding the focal field, which was divided into five concentric buffer circles at intervals of 100-meters.

### 2.3. DNA extraction, PCR and library preparation

A modified salt DNA extraction protocol (Sunnucks and Hales, 1996) was used to extract the genomic DNA of 732 adult spiders. Before performing DNA extractions, individual spiders were surface sterilized with 70% ethanol and washed three times with double distilled sterile water. Three lycosid individuals collected from the same field were pooled to perform a single DNA extraction. All the genomic DNA was kept at −80 °C until it was needed. Using the bacterial V3–V4 barcode region, 16S ribosomal RNA (rRNA) was amplified with the “338” forward primer (5′-ACTCCTACGGGAGGCAGCA-3′) and the “806” reverse primer (5′- GGACTACHVGGGTWTCTAAT-3′). The PCR settings were denaturation at 95°C for 5 min; 25 cycles of 95°C for 30 s, 50°C for 30 s, and 72°C for 40 s; and final extension at 72°C for 7 min. To purify the successful PCR products, VAHTSTM DNA magnetic beads was used to remove primers, dimers, salts, and deoxynucleoside triphosphates (dNTPs). Equally molar DNA libraries were prepared for pair-end sequencing using Illumina NovaSeq 6000 (Illumina, San Diego) platform.

### 2.4. Bioinformatics

Raw sequence data were primarily filtered by Trimmomatic (version 0.33) based on the quality of a single nucleotide (Bolger et al., 2014). Identification and removal of primer sequences were performed by Cutadapt (version 1.9.1) (Martin, 2011). Reads were demultiplexed, and paired-end reads were merged using USEARCH (version 10) (Edgar, 2010) followed by chimera removal using UCHIME (version 8.1) (Edgar et al., 2011). High-quality reads were obtained after removing chimeras. To analyze the microbial diversity information of the samples, clean tags were grouped at a 97% sequence similarity using USEARCH in QIIME. Different operational taxonomic units (OTUs) were obtained; then classified and annotated using the SILVA (bacteria) databases.

### 2.5. Data analyzes

To compare the bacterial composition in spider guts, and to investigate how different environmental variables (local field-scale and landscape scale) influence the gut microbial assemblages, all statistical analyses was conducted in R software (version 3.6.3). Differential abundance analyses were carried out at order level using R package “DESeq2” which allows identification of differentially abundant taxa between local field-scale groups: pesticide use (conventional vs. organic) and crop identity (Chinese cabbage vs. cauliflower). Multivariate orthogonal partial least squares discriminant analyses (OPLS-DA) were performed on the normalized log-transformed OTU abundance of bacterial orders considering as factor the combination of local field-scale groups. OPLS-DA was carried out with the R package “rolps”. The *p-*values and value of fold changes of differentially abundant taxa, and results of OPLS-DA were plotted together.

A distance-based redundancy analysis (dbRDA) model of Shannon diversities and abundances, based on Euclidean distances, was used to unveil the relationships between gut microbes of spiders with both local field-scale groups and landscape gradients. The community matrix was Hellinger transformed before performing the dbRDA. This transformation is often used in zero and one inflated community datasets because it downweighs variables with low counts and many zeros. To test the collinearity among each of the dbRDA model predictors, we used variation inflation factors (VIFs) method (James et al., 2013). Because environmental factors with VIF > 10 had collinearity with other environmental variables, they did not significantly contribute to the model's variance and were excluded from our final model. Additionally, an ANOVA-like permutation (999) test was used to determine the significance of dbRDA models and each environmental constraint (Legendre et al., 2011).

## 3. Results

### 3.1. Stability of gut bacterial community composition between pesticide uses, crop identity, and seasons

**Figure 2:**
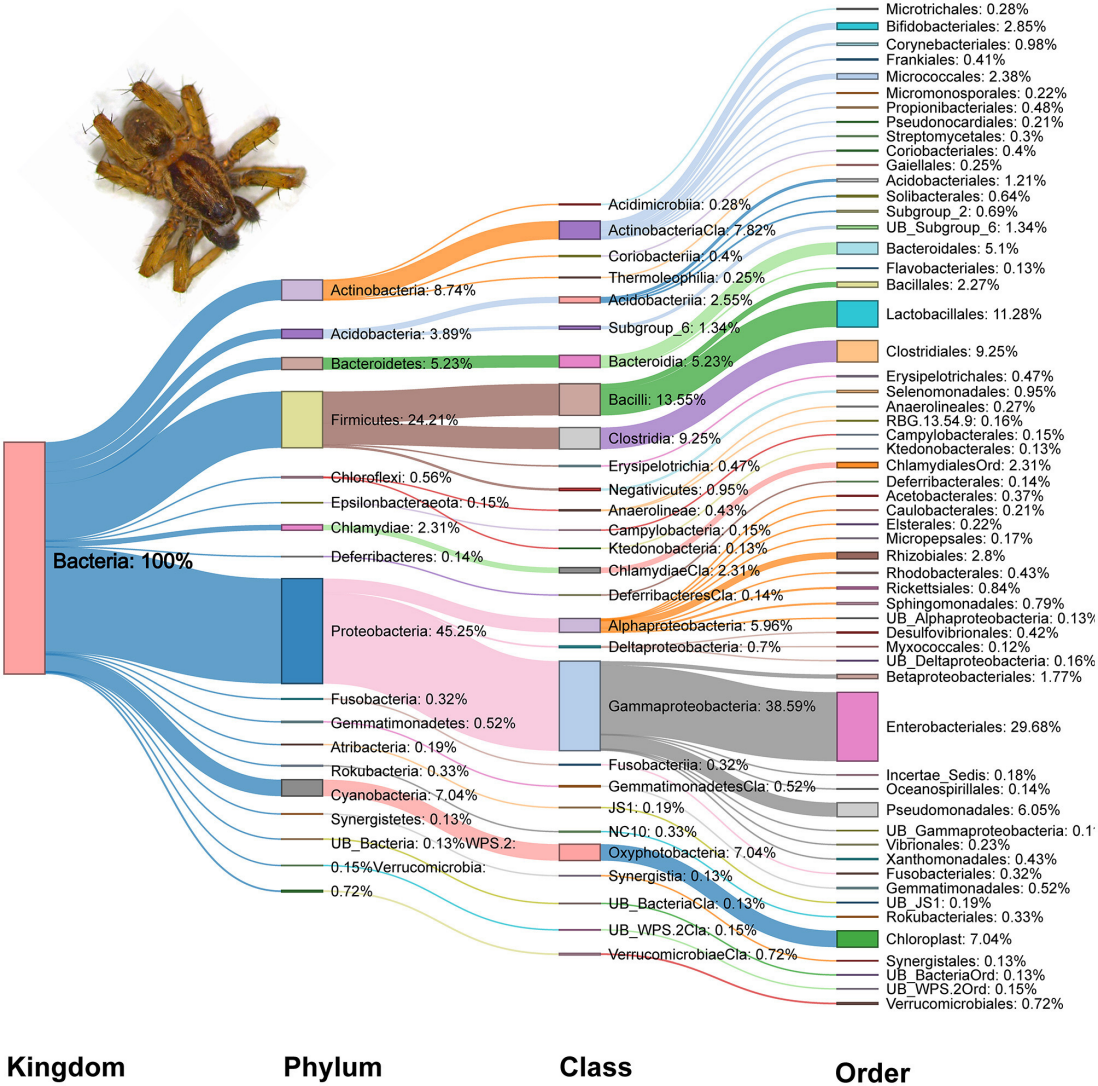
Sankey diagram of relative abundance (%) of dominant bacterial taxa (relative abundance ≥0.1%) detected in the gut of spiders.

At a 97 percent sequence alignment cutoff rate, Illumina Hiseq2500 sequencing generated a total of 15,911,790 (Bacteria-16S:V3+V4) clean paired reads, classified into 1,552 bacterial OTUs. A total of 27 phyla, 63 classes, 148 orders, 282 families, and 589 genera were identified. Among the gut bacterial communities, the dominant phylum was Proteobacteria (45.25%), followed by Firmicutes (24.21%), Actinobacteria (8.74%), Cyanobacteria (7.04%), and Bacteroidetes (5.23%) (Figure 2). Gammaproteobacteria (38.59%) was the most abundant class, followed by Bacilli (13.55%) and Clostridia (9.25%). Similarly, Enterobacteriales (29.68%) has the highest representation in the overall gut bacterial community composition at the order level, followed by Lactobacillales (11.28%) and Clostridiales (9.25%). In the phylum Proteobacteria, Gammaproteobacteria and Enterobacteriales were the most abundant class and order, respectively. Similarly, Bacilli and Lactobacillales were the dominant order and class, respectively in the phylum Firmicutes (Figure 2).

**Figure 3:**
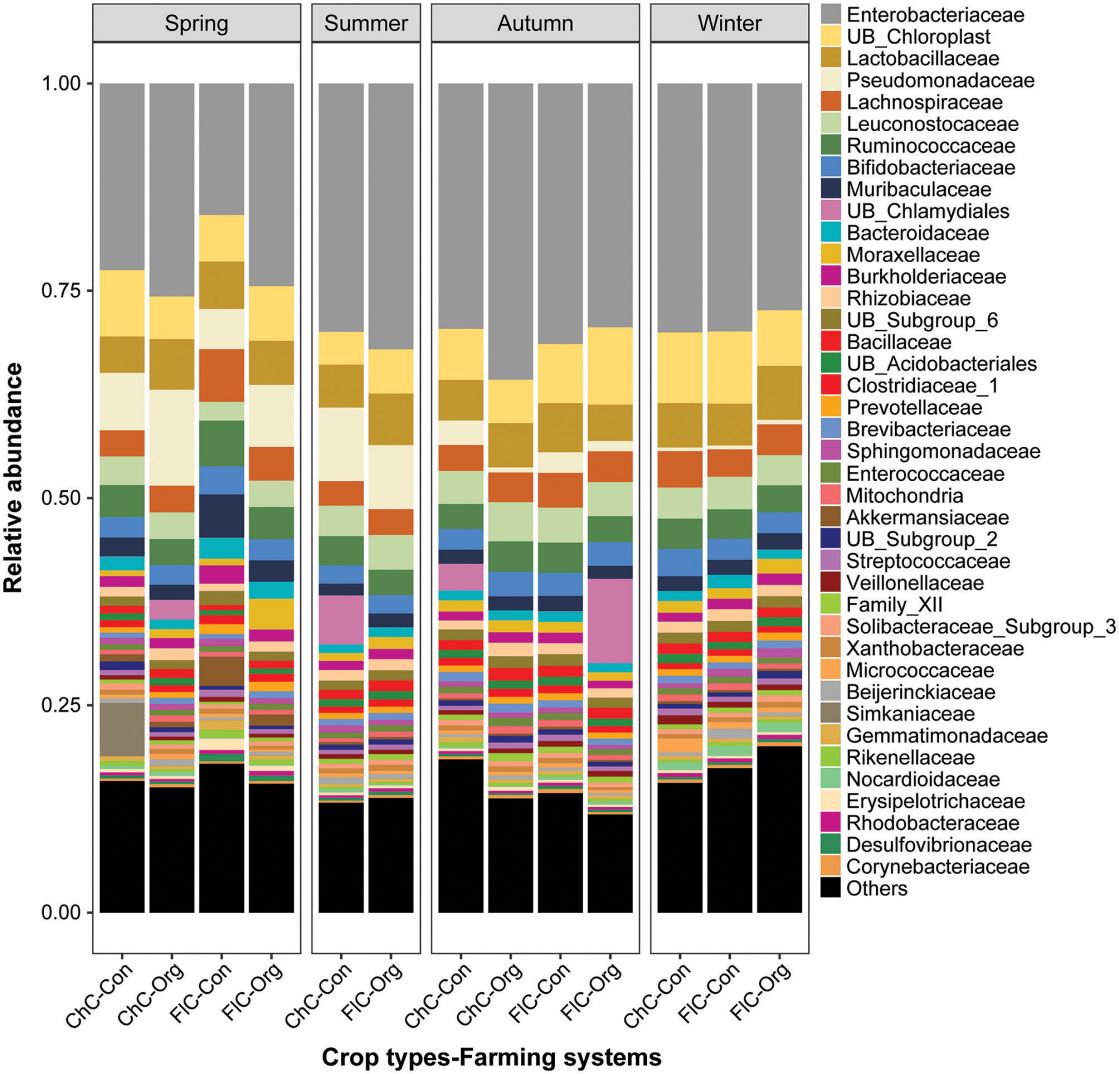
Relative abundance (%) of bacterial top 40 families detected in the gut of spiders. Spiders were captured from different brassica crop type (Chinese cabbage vs. cauliflower) fields managed under different farming systems (conventional vs. organic) across four seasons. Here, “ChC”, “FlC”, “Con”, and “Org” represent Chinese cabbage, cauliflower, conventional and organic respectively.

From the perspective of overall community composition, there was strong similarity across seasons, different pesticide practices, and crop identities. Microbiomes were dominated by Proteobacteria and Firmicutes phyla of bacteria, followed by Actinobacteria, Cyanobacteria, Bacteroidetes and Acidobacteriia (Supplementary Figure S1a). At the class level, Gammaproteobacteria and Bacilli represent the highest proportion of bacteria, followed by Clostridia, Actinobacteria, Oxyphotobacteria and Alphaproteobacteria (Supplementary Figure S1b). Furthermore, there were no changes to the dominance of the Enterobacteriales, Lactobacillales, Clostridiales, Chloroplast, Pseudomonadales, and Bacteroidales orders (Supplementary Figure S1c). Similarly, the Enterobacteriaceae, Chloroplast, Lactobacillaceae, Pseudomonadaceae, Lachnospiraceae, Leuconostocaceae, and Ruminococcaceae families were dominant regardless of the different farming systems, crop types and seasons (Figure 3).

### 3.2. Effects of pesticide uses and crop identity on gut bacterial community composition

**Figure 4:**
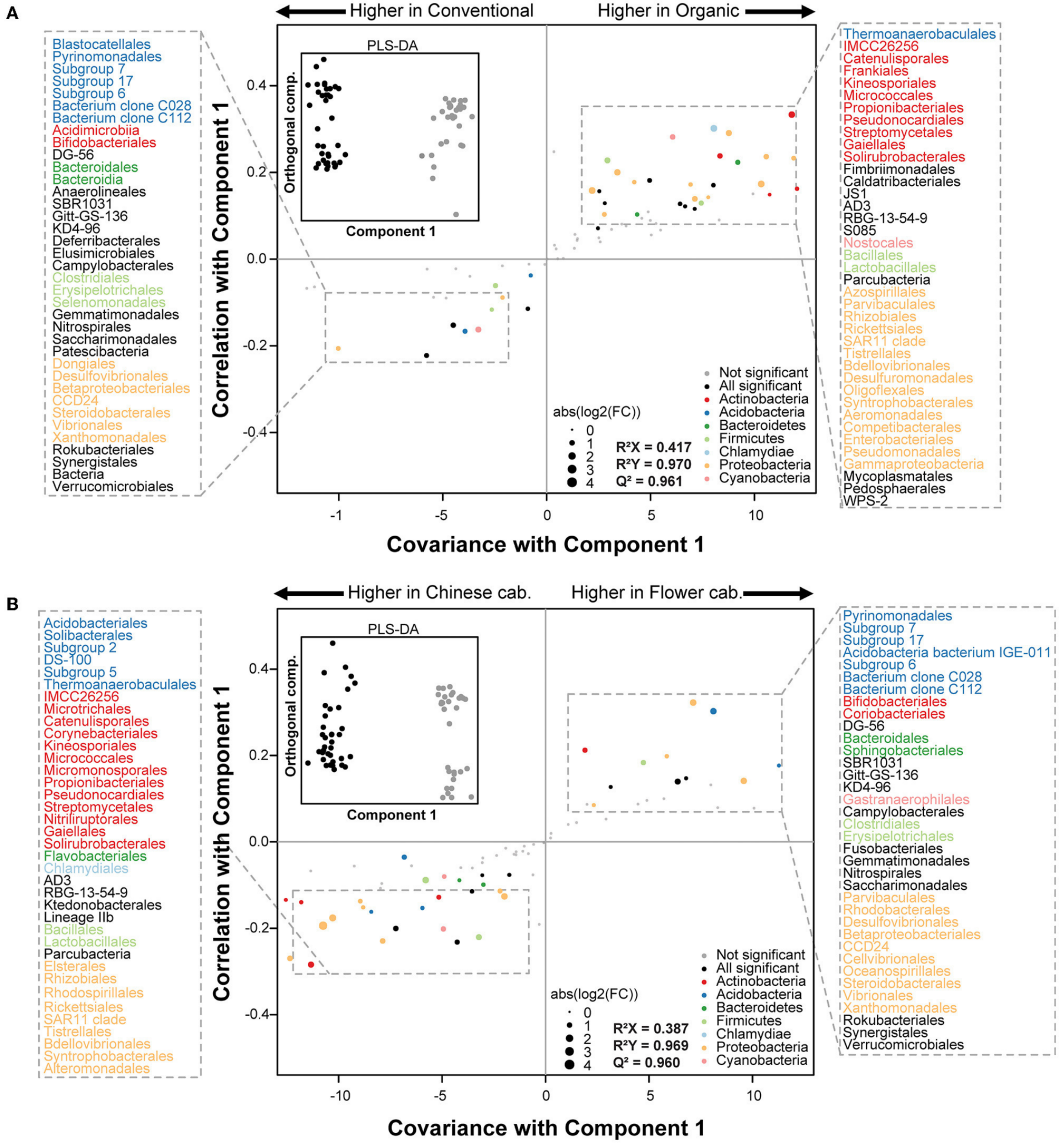
Gut microbiome changes of spiders between **(A)** pesticide uses (conventional vs. organic) and **(B)** crop identity (Chinese cabbage vs. cauliflower) in spring. The inside black-line boxes show the orthogonal partial least squares discriminant analyses (OPLS-DA) performed on the relative abundance of 148 bacterial orders. S-plots was generated in the main boxes from the results of OPLS-DA and differential abundance analyses. Each point shows the covariance (x-axis) and correlation (y-axis) from the predictive components of OPLS-DA model. Size of each point shows the value of fold change (FC) obtained from differential abundance analyses. Orders that were not robustly significantly (padj > 0.01) different between pesticide uses and crop identities are plotted in gray. Significant (padj < 0.01) families belonging to the top phyla (overall relative abundance >2%) are plotted in color and significant (padj < 0.01) families belonging to other phyla (overall relative abundance <2%) are plotted in black. padj corresponds to the *p*-value adjusted for multiple correlation testing using the Benjamini–Hochberg method.

A differential abundance analyses and OPLS-DA was performed to identify the bacterial orders that contributed the most to the variance in the gut of spiders collected from different pesticide uses and crop identities in different season. The overall gut bacterial composition was clearly different between different pesticide uses and crop identities at all four seasons (see inserted boxes of Figure 4, Supplementary Figures S2–S4).

A discriminant analyses on spider samples collected from the different pesticide uses and different crop identities revealed strongest differences in the relative abundance of gut bacterial taxa during spring (Figure 4) and autumn (Supplementary Figure S2). In spring, the 76 gut bacterial orders contributed the most in discriminating the pesticide uses (conventional verses organic) (Figure 4A), and 73 orders contributed to discriminating the crop identities (Chinese cabbage verses cauliflower) (Figure 4B). The most of differentially abundant orders between pesticide uses mainly belonged to the Acidobacteria (e.g., Blastocatellales, Pyrinomonadales, and Thermoanaerobaculales), Actinobacteria (e.g., Acidimicrobiia, Bifidobacteriales, Catenulisporales, Frankiales, Kineosporiales, Micrococcales, Propionibacteriales, Pseudonocardiales, and Streptomycetales), Firmicutes (e.g., Clostridiales, Erysipelotrichales, Selenomonadales, Bacillales, and Lactobacillales) and Proteobacteria (e.g., Dongiales, Desulfovibrionales, Betaproteobacteriales, Steroidobacterales, Azospirillales, Parvibaculales, Rhizobiales, Enterobacteriales, Pseudomonadales, and Gammaproteobacteria) phyla (Figure 4A). Similarly, during spring, the differences of gut bacterial orders concerning the crop identities were also exclusively assigned to the Acidobacteria (e.g., Acidobacteriales, Solibacterales, Thermoanaerobaculales, and Pyrinomonadales), Actinobacteria (e.g., Microtrichales, Catenulisporales, Corynebacteriales, Kineosporiales, Micrococcales, Pseudonocardiales, Bifidobacteriales, and Coriobacteriales), Firmicutes (e.g., Bacillales, Lactobacillales, Clostridiales, and Erysipelotrichales) and Proteobacteria (e.g., Elsterales, Rhizobiales, Rhodospirillales, Rickettsiales, Tistrellales, Parvibaculales, Rhodobacterales, Desulfovibrionales, and Betaproteobacteriales) phyla (Figure 4B).

In the autumn, discriminant analyses identified several significantly differentially abundant taxa in the gut of spiders collected from different pesticide uses and crop identities. The abundance of the 26 orders were significantly different between pesticide uses (organic verses conventional) fields (Supplementary Figure S2a), and 31 bacterial orders contributed to the differentiation between crop identities (Chinese cabbage verses cauliflower) (Supplementary Figure S2b). Differences concerned between pesticide uses almost exclusively assigned to the Acidobacteria (e.g., Blastocatellales, Subgroup 7, and Thermoanaerobaculales), Actinobacteria (e.g., Kineosporiales, Pseudonocardiales, and Streptomycetales) and Proteobacteria (e.g., Acetobacterales, Caulobacterales, Rhodobacterales, Enterobacteriales, and Alphaproteobacteria) phyla (Supplementary Figure S2a). The spider collected from Chinese cabbage had the significantly higher abundance of phyla Acidobacteria (e.g., Subgroup 7 and soil Bacterium clone C028), Actinobacteria (e.g., Corynebacteriales and Pseudonocardiales), Bacteroidetes (Bacteroidales, Chitinophagales, and Sphingobacteriales) and Proteobacteria (e.g., Dongiales, SAR11 clade, and Vibrionales) (Supplementary Figure S1b). On the other hand, the guts of spider collected from cauliflower was significantly differentially abundant with phyla Actinobacteria (e.g., Frankiales, Kineosporiales, and Micromonosporales) and Proteobacteria (e.g., Azospirillales, Elsterales, and Rickettsiales) (Supplementary Figure S2b).

In winter, a total of 29 bacterial orders (e.g., Acidobacteriales, Solibacterales, Pyrinomonadales, Catenulisporales, Cytophagales, Bacillales, Caulobacterales, and Elsterales) were significantly differentially abundant in organic fields compared with the gut of spiders collected form conventionally managed fields were significantly differentially dominated with 14 bacterial orders (e.g., Kineosporiales, Chloroplast, Rickettsiales and Bdellovibrionales) (Supplementary Figure S3a). Moreover, 13 bacterial orders (e.g., Corynebacteriales, Frankiales, Actinobacteria and Sphingomonadales, Desulfuromonadales, and Myxococcales) were significantly differentially dominant in the guts of spiders collected from cauliflower fields compared with samples collected from Chinese cabbage had 12 significantly differentially abundant bacterial orders (e.g., Bifidobacteriales, Coriobacteriales, Sphingobacteriales, Clostridiales, and Micropepsales) (Supplementary Figure S3b). In summer, however, the total of 20 gut bacterial orders (e.g., Aminicenantales, Streptosporangiales, Chloroplast, Lactobacillales, Acetobacterales, and Rickettsiales) were significantly higher in spiders collected from cauliflower fields under organic management practices, whilst only five gut bacterial orders (e.g., Acetothermiia, Propionibacteriales, Sphingobacteriales, and Oligoflexales) were significantly differentially abundant in spiders captured from Chinese cabbage field under conventional management practices (Supplementary Figure S4). These results of discriminant analyses highlighted that certain gut microbes of spiders showed a clear divergence between pesticide uses and crop identities across different seasons.

### 3.3. Local scale groups and landscape gradients as determinants of gut microbial assemblages

**Figure 5:**
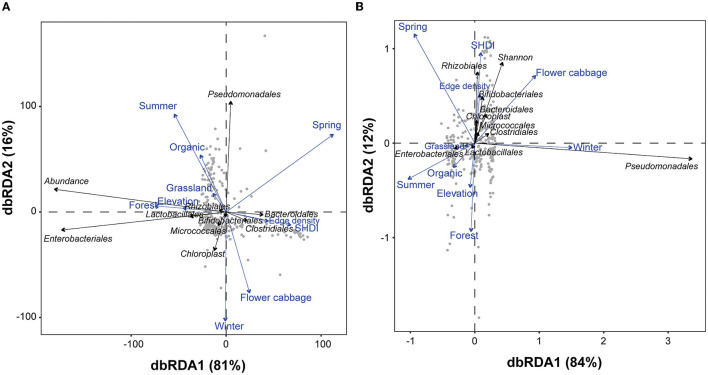
Distance based redundancy analyses (dbRDA) illustrating the associations of overall and top ten bacterial taxa with various environmental factors in terms of **(A)** abundance and **(B)** diversity in the gut of spiders. For each variable, the arrows' length and orientation indicate the magnitude of explained variance. The association between bacterial taxa detected in spider guts and explanatory factors represented by the perpendicular distance between them (below-90° = positive association and above-90° = negative association). The association is larger when the perpendicular distance is less. The “SHDI” represents the Shannon-Wiener diversity index (SHDI) of different surrounding vegetation.

The dbRDA analyses revealed effects of landscape features, seasons, crop types and farming systems on the OTU composition of the spiders' gut flora, both when the entire gut microbiome and a subset of the most numerous nine orders were considered (Figure 5). The differences in the OTU composition of microbes detected in the gut of spiders were significant between local- and landscape-factors across different seasons (dbRDA model permutation test). Only the “spatial scales” variable (i.e., differing sized radii around the focal field) was found to be redundant (having VIF > 10) in the final dbRDA models both in those based on the abundance and Shannon-diversity of gut microbes, therefore it was removed. The first two axes in the final dbRDA model explained 96% of total variability in the assemblage structure of gut microbes based on their abundance and 95% based on their OTUs' Shannon-diversity. Consistent with the PERMANOVA results, the permutation of dbRDA also showed that all the local and landscape factors significantly explained the variability observed in the assemblage structure of both abundance and Shannon-diversity of the gut microbes in spiders.

The higher proportions of forests, grasslands and edge density accounted for significantly higher fractions of the variability in assemblages of gut microbes in terms of both abundance and Shannon-diversity (Table 1). The compositional diversity of landscape (SHDI), however, accounted for the higher fractions of the variability in terms of the OTUs' Shannon-diversity (OTUs' Shannon-diversity—*F* = 2.000, *p* = 0.043). At local field scale, the community structure of the gut microbes was significantly influenced by the crop identity, pesticide use, seasons, and elevation gradients both when the abundance and the OTUs' Shannon-diversity was considered. The seasons and crop identity, on the other hand, accounted for explaining the highest fractions of the variability in gut microbe assemblages in term of both abundance and Shannon-diversity (Table 1).

**Table 1:** ANOVA table to test the significance of each predictor in RDA model explaining the variance of bacterial communities in the gut of spiders.

**Variables**	**Abundance**		**Diversity**	
	**F**	***P*** **-value**	**F**	***P*** **-value**
RDA models	40.012	0.001	58.415	0.001
**Land use variables**				
Edge density	15.108	0.001	7.808	0.001
SHDI	1.064	0.326	2.000	0.043
Forest	25.961	0.001	7.926	0.001
Grassland	3.706	0.02	4.260	0.006
**Other variables**				
Crop identity	47.005	0.001	58.740	0.001
Pesticide use	19.935	0.001	5.979	0.001
Season	84.635	0.001	162.692	0.001
Elevation	33.436	0.001	9.358	0.001

The overall gut microbial abundance and the abundances of Enterobacteriales, Lactobacillales, Rhizobiales were higher in fields surrounded with high proportion of forest, and grassland located at higher elevation during autumn season. Conversely, the abundance of Bacteroidales and Clostridiales gut microbes was strongly associated with higher edge density and higher SHDI. Cauliflower fields had higher abundance of Bifidobacteriales, Micrococcales and Chloroplast microbes during winter (Figure 5A). The overall Shannon-diversity and the Shannon-diversity of Rhizobiales, Bifidobacteriales, Bacteroidales, Chloroplast, Clostridiales and Micrococcales were strongly associated with the cauliflower crop type and the higher edge density, as well as the higher landscape compositional diversity (SHDI) during spring season. The Shannon-diversity of Pseudomonadales in the gut of spiders was higher during winter season. Conversely, the Shannon-diversity of Lactobacillales and Enterobacteriales in the gut of spiders was strongly linked to the organic farming systems with higher proportions of grasslands and forest patches, and higher elevation during summer season (Figure 5B).

## 4. Discussion

Here, we have demonstrated that variations in different local environmental conditions and the composition of the surrounding landscape can distinctly alter the gut microbiota of spiders. This study provides powerful insight into the plasticity of bacterial diversity and abundance in the gut of the most abundant predators in agricultural crops and demonstrates for the first time the effects of seasons, crop identity, pesticide use, and surrounding landscape structure on the gut microbiota. In this study, we detected 27 phyla, 64 classes, 148 orders, 282 families, and 589 genera of bacteria. The dominant bacterial taxa (such as Proteobacteria, Firmicutes, Actinobacteria, Cyanobacteria, Bacteroidetes, and Acidobacteria) represents more than 80% of the entire gut bacterial community of spiders. Proteobacteria have been demonstrated to synthesize vital micronutrients, including vitamin K2, interact with the immune system to stimulate the generation of immune cells and modulate immune function within the gut environment, produce short-chain fatty acids that play a crucial role in energy metabolism and maintaining gut health (Ley et al., 2006; Round and Mazmanian, 2009; LeBlanc et al., 2013). Similarly, Firmicutes contribute to the breakdown of complex carbohydrates in the insect gut to facilitate energy metabolism and gut health. They also trigger the production of immune effectors such as antimicrobial peptides, mediate symbiotic relationships with other gut microbes to enhance nutrient cycling and other physiological processes, and protect against harmful pathogens either by direct antagonism or by modulating the immune response (Shin et al., 2011; Chaston et al., 2014; Ceja-Navarro et al., 2015). We have clearly demonstrated that the relative abundance of these functionally important gut bacterial taxa were distinctly influenced by different local field-scale and landscape-scale variables.

Prior studies showed that composition of diet is closely related to gut microbiota of arthropods (Gupta and Thorsteinson, 1960; Dong et al., 2018; Yang et al., 2020). In our study, the composition of the gut microbiota was substantially discriminated by crop identity, which is most likely underpinned by the dependency of the spiders' diet on the same factor (Saqib et al., 2021). Indeed, spiders are known as generalist predators which prey on a diverse range of herbivores as well as on other predator species (intraguild predation) (Hambäck et al., 2021; Saqib et al., 2021), depending on availability of these in the habitat. On the other hand, plant-eating insects gather their diverse gut microbial population by feeding on different kinds of crops, which, in turn, is responsible for the differences in gut microenvironment of these herbivores. Thus, the potential pool of microbes for horizontal bacterial transfer changes with the environment in which herbivores feeds and this can lead to the variation in gut microbial composition of the associated predators. For example, the gut bacterial community structure of *Monochamus alternatus, Psacothea hilaris* (Coleoprera: Cerambycidae), *Rhodococcus* and *Achromobacter* genera have a strong link with natural diet samples, and the *Buttiauxella* and *Kluyvera* genera showed a significant correlation with artificial diet samples (Kim et al., 2017). Wild crickets, *Teleogryllus oceanicus* (Orthoptera: Gryllidae) have five bacterial phyla (*Cyanobacteria, Fusobacteria, Lentisphaerae, Planctomycetes*, and *Synergistetes*) that were not detected in lab-reared crickets (Ng et al., 2018). In addition, some Lepidopteran insects such as *Thaumetopoea pityocampa* and *Plutella xylostella* also showed an interplay of strong interactions between the insect gut bacterial community and host plants (Strano et al., 2018). The abundance of Proteobacteria phylum was also found to be less in the larvae of *T. pityocampa* feeding on *Pinus halepensis* (Pinales: Pinaceae) than those collected on *Pinus nigra* subsp. *laricio* or *Pinus pinaster* (Strano et al., 2018). Our work, however, is pioneer in this regard because no similar studies are found on spiders. The above findings suggested that generalist predators foraging on diverse prey range (including herbivores as well as intraguild prey) which feed on different crop plants will eventually determine the assemblage structure of predator's gut microbiota.

Similar to the impacts of the host crops, there were considerable effects of different pesticide uses on discriminating the assemblages of gut microbial taxa in spiders. It is widely reported that increasing organic farming would lead to higher farmland biodiversity (Fuller et al., 2005; Schmidt et al., 2005; Batáry et al., 2011; Garratt et al., 2011). Plant species density has been reported to be higher in organic fields (both inside the crop fields and adjacent non-crop areas) than in conventional ones (Chateil et al., 2013), which may provide a range of resources, and thus contribute to the more diverse assemblages of arthropods. Conventional farms receive more synthetic herbicides to ensure weed control, resulting in lower plant species diversity (both within the crop fields as well as in adjacent areas), which ultimately has severe impacts on the diversity of inhabiting arthropods. Similarly, study have reported the variations in chemical composition of soil as well as plant metabolites (e.g., pH, organic matter, nutrients, and root exudates) between conventional and organic farming systems (Cubero-Leon et al., 2018; Armalyte et al., 2019). These changes of plant metabolites may also drive changes in metabolic structure of herbivores (Kešnerová et al., 2017; Yang et al., 2020), which might eventually influence the assemblages of gut microbiota of herbivores and their associated predators.

In agricultural land, where pesticides are applied the microbiome may also facilitate the development of insecticide resistance. Yet, little research effort has been made to examine how insect gut microbial populations affect resistance and biodegradation of pesticides in generalist predators. Kikuchi et al. (2012) showed that the gut symbiont *Burkholderia* mediates pesticide resistance in *Riptortus pedestris* (Hemiptera) and that this resistance may be horizontally transferred to other insects. Direct biodegradation of pesticides by gut microbiota, such as *R. pedestris* (Kikuchi et al., 2012) and *B. dorsalis* (Cheng et al., 2017), and immune modulation, in which microbiota induce the development of an innate immune response (Broderick et al., 2010), have been proposed as drivers of resistance development. Bacteria including *Bacillus aerophilus, Klebsiella pneumonia, Mycobacterium* sp, *Pseudomonas* sp. 1G, *Pseudoxanthomonas, Stenotrophomonas* sp., *Rhodococcus* sp. found to be involved in pesticide resistance, and biodegradation of neonicotinoids (Xia et al., 2018). Similarly, our study showed the effect of farming practices on the abundance and sensitivity of certain bacterial taxa in the gut of spiders, including Desulfuromonadales, Pseudomonadales, and Steroidobacterales. We also discovered that the sensitivity of these bacterial populations to farming practices is indicative of the significant impacts that different farming systems may have on these gut bacterial populations. However, detailed studies are required to reveal the complete underlying mechanisms of pesticide resistance in natural enemies and to highlight the critical roles of different genes and enzyme of gut microbes involved in the detoxification of pesticides.

The insect gut microbiota also changes with season (e.g., Ferguson et al., 2018). Pseudomonadales (belong to phylum Proteobacteria) were found to be dominant in spiders sampled during summer, whereas Lactobacillales (phylum Firmicutes), and Enterobacteriales (phylum Proteobacteria) were dominant in the bacterial profile of samples collected during autumn. Indeed, Duguma et al. (2017) indicated that the Proteobacteria abundance in *Culex* mosquitos may be associated with factors such as temperature. Overall, our results showed that spider samples collected in summer had the least diverse microbiome in terms of diversity compared to samples collected in spring, autumn and winter. Research shows that high temperature could affect the growth of plants, and the growth and development of insects, even alter the insect's feeding behavior, and indirectly can influence the assemblages of gut microbiota of arthropods (Sepulveda and Moeller, 2020).

To date, surrounding habitat and the ecological conditions shaping the microbial community in the gut of generalist predators has received comparatively little attention. In our study, we showed that all the landscape variables we investigated significantly linked to the variations observed in the assemblages of the gut microbiota of spiders; particularly varying landscape composition diversity (SHDI), and the proportions of forest and grassland patches, as well as edge density, were the factors which showed significant influences. Several studies reported that varying proportions of different land uses and edge density (Landis et al., 2000; Perović et al., 2015; Gallé et al., 2018), as well as the landscape compositional diversity (Zhang et al., 2021) in the surrounding landscape, may alter the assemblage patterns of farmland biodiversity, which impact may cascade through various trophic levels and drive the distribution of microbiomes of spiders. In a characterization study of several insect species and their associated gut microbiota, the relative occurrences of microbes were found to vary according to the surrounding environmental habitats of the insects (Yun et al., 2014). Overall, these results suggest that the surrounding environment that spiders are exposed to, including varying proportions of different land uses, environmental conditions, or prey range, may highly affect their gut microbial assemblages.

## 5. Conclusions

In summary, our study findings suggest that the diverse environmental exposures at local and broad landscape levels have distinctive effects on the assemblages of gut microbiota of spiders. Our results underscore the potential influence of key environmental factors on specific gut microbes (such as Proteobacteria and Firmicutes), and emphasize the need for future laboratory-based investigations for understanding their functional role in growth, development, nutrition and reproduction of spiders. Our study revealed that several bacterial species inhabit the gut of spiders, potentially conferring a fitness advantage through enhanced resistance to pesticides. Nevertheless, further investigations are necessary to comprehensively elucidate the intricate mechanisms governing pesticide resistance in predatory arthropods. While it is hard to determine the origin of microbes inside the body of generalist predators, it is paramount to assess or establish whether these microbes were directly acquired from the environment or a result of an indirect acquisition through predation on varying prey species or vertical transmission from the mother spider. This study, however, highlights the possible complex interplay between host, its gut microbial community, and the key environmental factors (at various local- and landscape-scales), and identifies the key bacteria taxa to target in future investigations.

## Data availability statement

The datasets for this study can be found in online repositories. The name of the repositories and accession number can be found below: https://www.ncbi.nlm.nih.gov/, SAMN34395390 to SAMN34395539, SAMN34395697 to SAMN34395796.

## Author contributions

HS, MY, SY, and GG conceived and designed the experiments. HS, KA, and LS conducted field sampling and lab experiments. HS performed the data analyses and prepared figures and tables. HS, GP, MG, and GG interpreted the data and wrote the paper. PL, SY, and GP assisted in the data analyses. All authors revised the final version and gave their approval for submission.
