# Supplementary material for: Gut microbiota assemblages of generalist predators are driven by local- and landscape-scale factors
Source: Front Microbiol. 2023 May 15;14:1172184. doi: 10.3389/fmicb.2023.1172184 (PMC10225636; doi:10.3389/fmicb.2023.1172184)
Supplement: Supplementary file 1 [file Data_Sheet_1.docx]

*Supplementary information*

**Gut microbiota assemblages of generalist predators are driven by local- and landscape-scale factors**

Hafiz Sohaib Ahmed Saqib ^1,2,3,4,†^, Linyang Sun ^1,3,4,†^, Gabor Pozsgai ^1,3,4,5^, Pingping Liang ^6^, Mohsan Ullah Goraya ^7^, Komivi Senyo Akutse ^8^, Minsheng You ^1,3,4^, Geoff M. Gurr ^1,3,4,9*^, Shijun You ^1,3,4*^

^1^ State Key Laboratory for Ecological Pest Control of Fujian and Taiwan Crops, Institute of Applied Ecology, Fujian Agriculture and Forestry University, Fuzhou 350002, China

^2^ Guangdong Provincial Key Laboratory of Marine Biology, College of Science, Shantou University, Shantou 515063, China

^3^ Joint International Research Laboratory of Ecological Pest Control, Ministry of Education, Fuzhou 350002, China

^4^ Ministerial and Provincial Joint Innovation Centre for Safety Production of Cross-Strait Crops, Fujian Agriculture and Forestry University, Fuzhou 350002, China

^5^ Ce3C - Centre for Ecology, Evolution and Environmental Changes, Azorean Biodiversity Group, CHANGE – Global Change and Sustainability Institute, University of the Azores, Faculty of Agricultural Sciences and Environment, Angra do Heroísmo, Terceira, Açores, Portugal

^6^ Center for Infection and Immunity, Guangdong Provincial Engineering Research Center of Molecular Imaging, Guangdong Provincial Key Laboratory of Biomedical Imaging, the Fifth Affiliated Hospital, Sun Yat-sen University, Zhuhai, Guangdong, 519000, China

^7^ Guangdong Provincial Key Laboratory of Infectious Diseases and Molecular Immunopathology, Shantou University Medical College, Shantou, 515041, China

^8^ Plant Health Theme, International Centre of Insect Physiology and Ecology, Nairobi P.O. Box 30772-00100, Kenya

^9^ Gulbali Institute, Charles Sturt University, Orange, NSW 2800, Australia

^†^ These authors have contributed equally to this work

^*^ Corresponding authors e-mails and telephone numbers: [ggurr@csu.edu.au](mailto:ggurr@csu.edu.au) (+61 2 63657551); [sjyou@fafu.edu.cn](mailto:msyou@fafu.edu.cn) (+86 591 83844953)


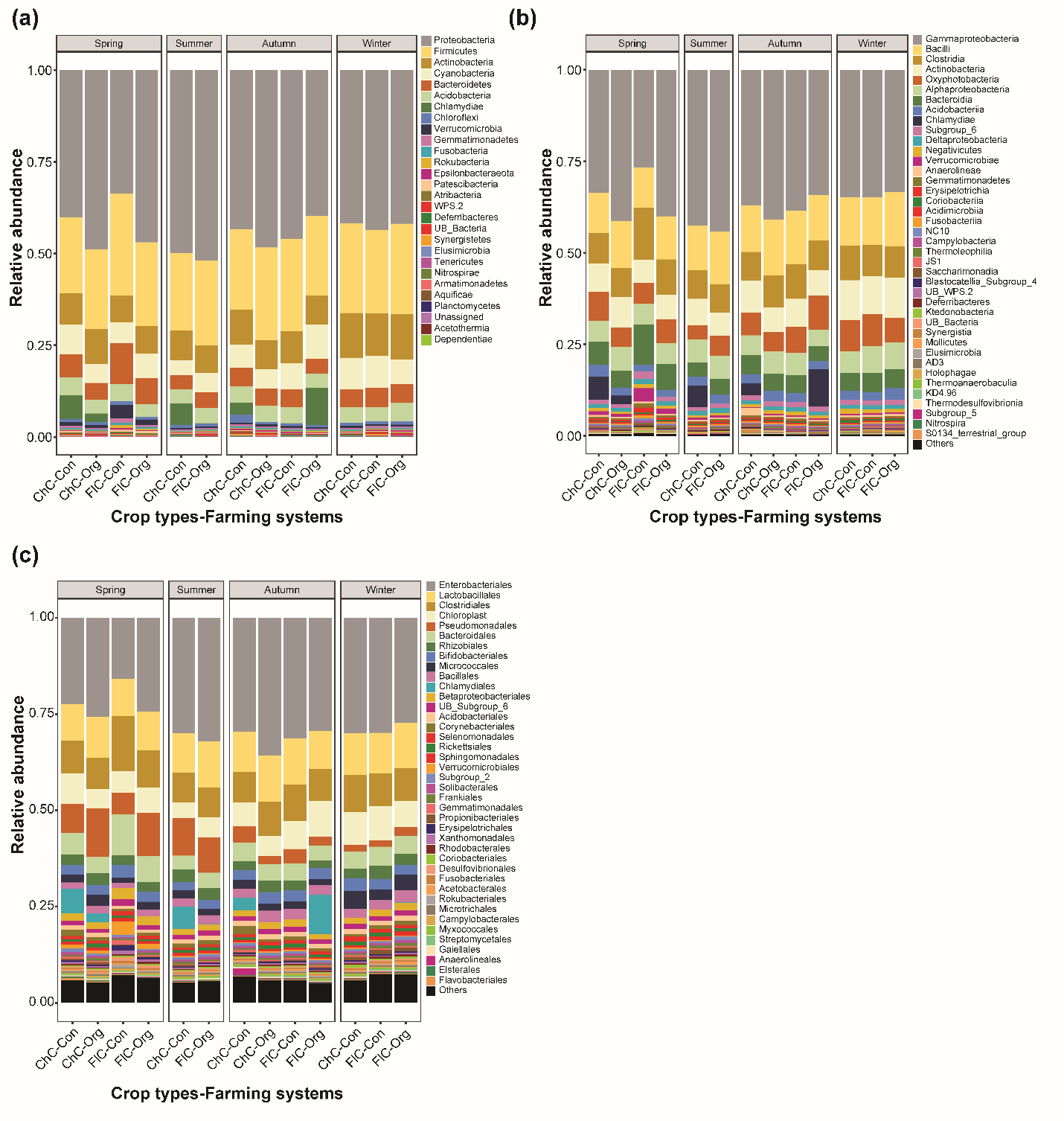


**Figure S1** Relative abundance (%) of bacterial (a) phylum, top 40 (b) classes, and (c) orders detected in the gut of spiders. Spiders were captured from different brassica crop type (Chinese cabbage vs cauliflower) fields managed under different farming systems (conventional vs organic) across four seasons. Here, “ChC”, “FlC”, “Con” and “Org” represent Chinese cabbage, cauliflower, conventional and organic respectively.


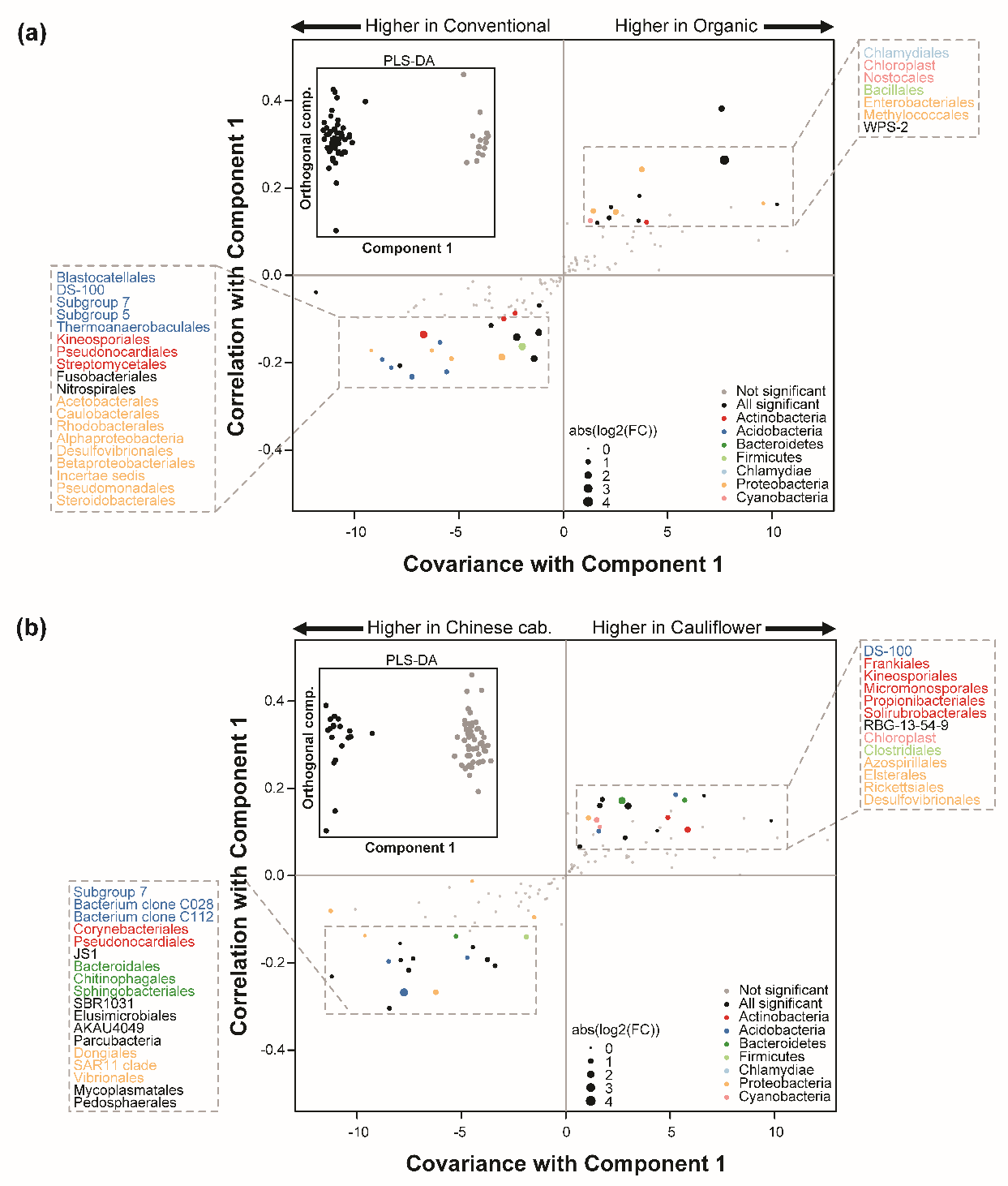


**Figure S2** **Gut microbiome changes of spiders between (a) pesticide uses (conventional versus organic) and (b) crop identity (Chinese cabbage versus cauliflower) in autumn.** The inside boxes black-line show the orthogonal partial least squares discriminant analyses (OPLS-DA) performed on the relative abundance of 148 bacterial orders. S-plots was generated in the main boxes from the results of OPLS-DA and differential abundance analyses. Each point shows the covariance (x-axis) and correlation (y-axis) from the predictive components of OPLS-DA model. Size of each point shows the value of fold change (FC) obtained from differential abundance analyses. Orders that were not robustly significantly (padj > 0.01) different between pesticide uses and crop identities are plotted in grey. Significant (padj < 0.01) families belonging to the top phyla (overall relative abundance > 2%) are plotted in color and significant (padj < 0.01) families belonging to other phyla (overall relative abundance < 2%) are plotted in black. padj corresponds to the *p*-value adjusted for multiple correlation testing using the Benjamini–Hochberg method.


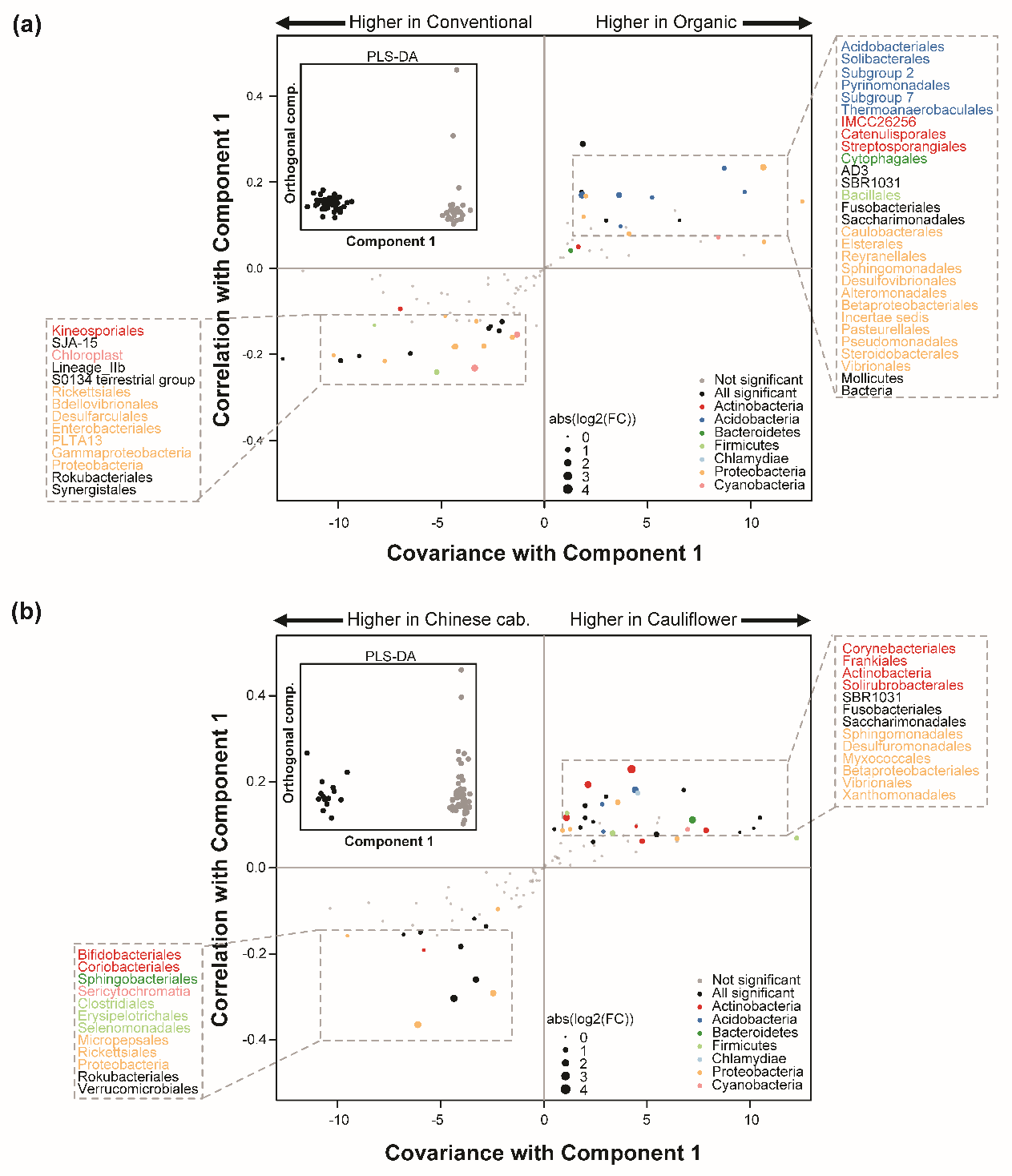


**Figure S3** **Gut microbiome changes of spiders between (a) pesticide uses (conventional versus organic) and (b) crop identity (Chinese cabbage versus cauliflower) in winter.** The inside black-line boxes show the orthogonal partial least squares discriminant analyses (OPLS-DA) performed on the relative abundance of 148 bacterial orders. S-plots was generated in the main boxes from the results of OPLS-DA and differential abundance analyses. Each point shows the covariance (x-axis) and correlation (y-axis) from the predictive components of OPLS-DA model. Size of each point shows the value of fold change (FC) obtained from differential abundance analyses. Orders that were not robustly significantly (padj > 0.01) different between pesticide uses and crop identities are plotted in grey. Significant (padj < 0.01) families belonging to the top phyla (overall relative abundance > 2%) are plotted in color and significant (padj < 0.01) families belonging to other phyla (overall relative abundance < 2%) are plotted in black. padj corresponds to the *p*-value adjusted for multiple correlation testing using the Benjamini–Hochberg method.


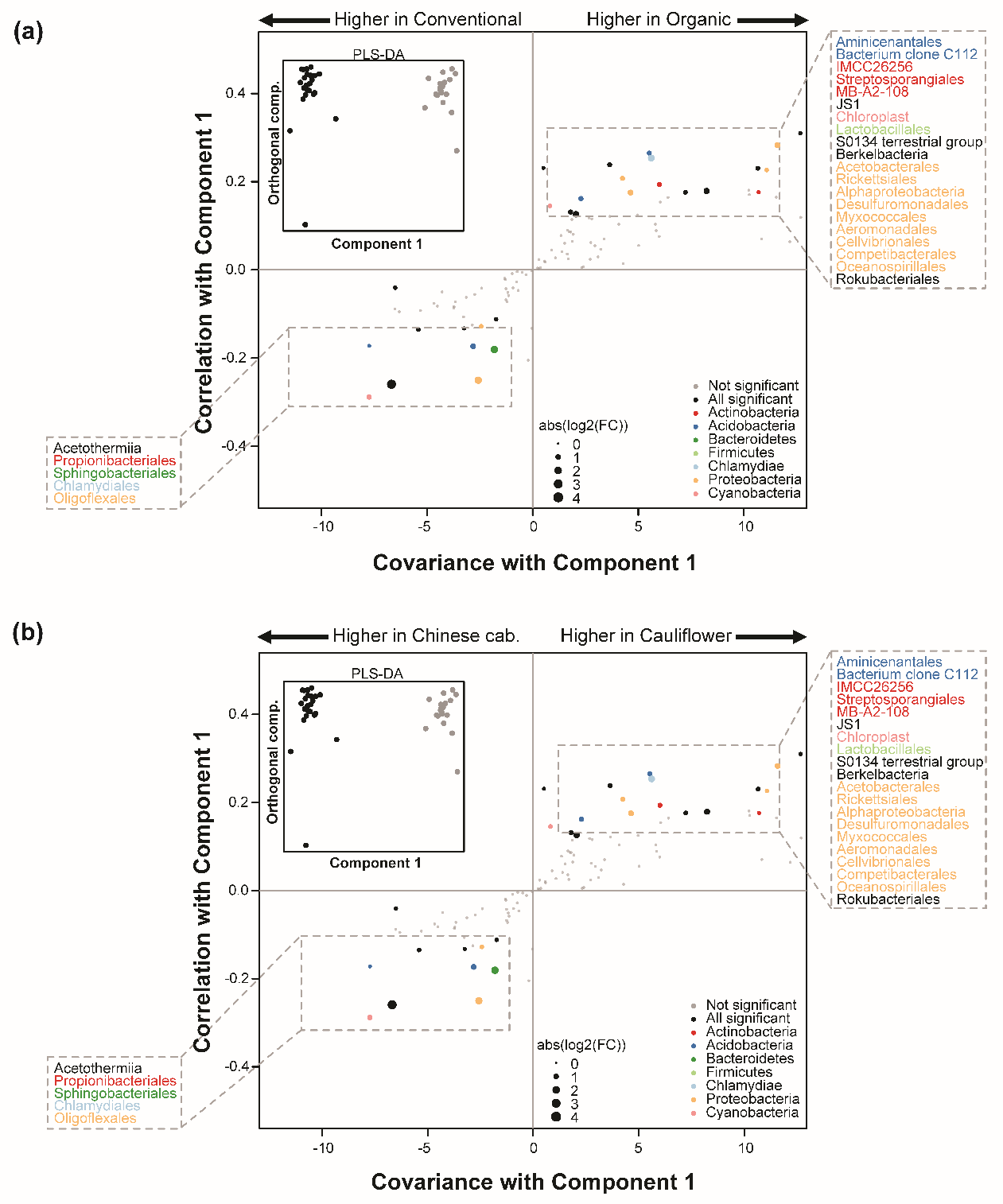


**Figure S4** **Gut microbiome changes of spiders between (a) pesticide uses (conventional versus organic) and (b) crop identity (Chinese cabbage versus cauliflower) in summer.** The inside black-line boxes show the orthogonal partial least squares discriminant analyses (OPLS-DA) performed on the relative abundance of 148 bacterial orders. S-plots was generated in the main boxes from the results of OPLS-DA and differential abundance analyses. Each point shows the covariance (x-axis) and correlation (y-axis) from the predictive components of OPLS-DA model. Size of each point shows the value of fold change (FC) obtained from differential abundance analyses. Orders that were not robustly significantly (padj > 0.01) different between pesticide uses and crop identities are plotted in grey. Significant (padj < 0.01) families belonging to the top phyla (overall relative abundance > 2%) are plotted in color and significant (padj < 0.01) families belonging to other phyla (overall relative abundance < 2%) are plotted in black. padj corresponds to the *p*-value adjusted for multiple correlation testing using the Benjamini–Hochberg method.
